# *Mycobacterium vaccae* Adaptation to Disinfectants and Hand Sanitisers, and Evaluation of Cross-Tolerance with Antimicrobials

**DOI:** 10.3390/antibiotics9090544

**Published:** 2020-08-27

**Authors:** Carla C. C. R. de Carvalho, Raquel Teixeira, Pedro Fernandes

**Affiliations:** 1 Department of Bioengineering, iBB–Institute for Bioengineering and Biosciences, Instituto Superior Técnico, Universidade de Lisboa, Av. Rovisco Pais, 1049-001 Lisbon, Portugal; raquel.cristina@tecnico.ulisboa.pt (R.T.); pedro.fernandes@tecnico.ulisboa.pt (P.F.); 2 DREAMS and Faculty of Engineering, Universidade Lusófona de Humanidades e Tecnologias, 1749-024 Lisbon, Portugal

**Keywords:** antibiotic, levofloxacin, teicoplanin, efflux pump, thioridazine, omeprazole, phenotypic adaptation, permeability, tolerance, resistance

## Abstract

*Mycobacterium vaccae* is being considered as an adjuvant to antituberculosis therapy, tested for the treatment of autoimmune diseases, and as an anti-depressive agent. This bacterium is ubiquitous in the environment and the widespread use of disinfectants and sanitisers may lead to its adaptation to these compounds. In the present study, *M. vaccae* cells adapted to these compounds mainly by making adjustments in their lipid composition and net surface charge. The modifications in the lipid composition led to changes in membrane permeability which resulted in increased tolerance towards levofloxacin, thioridazine, and omeprazole.

## 1. Introduction

The genus *Mycobacterium* belongs to the family Corynebacteriaceae which includes other highly efficient biodegraders and biocatalysts such as *Nocardia*, *Gordonia*, and *Rhodococcus* [1,2,3,4]. Its most known species, *Mycobacterium tuberculosis*, is responsible for the highest mortality caused by a single infectious agent. In 2018, an estimated 10.0 million people had tuberculosis (TB) and 1.4 million died worldwide [5]. Among the new cases, 3.4% had multidrug resistant TB or rifampicin-resistant TB.

*Mycobacterium vaccae* is a fast-growing nontuberculous mycobacterial species, initially considered non-pathogen. In 1996 it was first reported to cause invasive disease in humans, being responsible for cutaneous or pulmonary infections [6]. Since then, few nosocomial infections caused by *M. vaccae* have been reported [2,3,4]. However, it has been tested efficiently as an immunotherapeutic agent for the treatment of TB [7], autoimmune diseases [8], and to improve long-term survival and the quality of life of cancer patients [9,10].

Nontuberculous mycobacteria are ubiquitous in water and soil but can also be found in man-made structures such as water distribution systems [11]. The presence of long chain mycolic acids on the cell envelop of mycobacteria is responsible for the hydrophobicity, impermeability, growth characteristics, and tolerance to toxic compounds such as disinfectants and antibiotics [12,13,14,15].

We have previously studied the response of *Mycobacterium* sp. cells to organic solvents relevant for bioprocesses [16,17,18]. Additionally, we also studied *M. vaccae* adaptation to organic solvents and showed that the adapted cells increased tolerance toward the efflux pump inhibitors (EPIs) thioridazine and omeprazole, but were more susceptible to the antibiotics levofloxacin and teicoplanin when compared to non-adapted cells [19]. Major responses involved alterations at the lipid composition of the cellular membranes which were accompanied by changes in the zeta potential of the surface of the cells. Changes in the fatty composition of the phospholipids of the cellular membrane are a cell strategy to maintain the fluidity condition of the membrane, a mechanism known as homeoviscous adaptation [20,21].

The present study is aimed at assessing if *M. vaccae* cells adapt to disinfectants and if possible cross-adaptation mechanisms between exposure to disinfectants, EPIs, and antibiotics exist. Tolerance/resistance to disinfectants and sanitisers may be a serious health and economic burden. Co-resistance of disinfectant-resistant bacteria to antibiotics is known to occur with quaternary ammonium compounds and peroxides as result of resistance genes being located on transmissible plasmids, and within conserved regions of integrons, which also harbour multiple antibiotic resistance genes [22]. Genes encoding for efflux pumps capable of expelling many quaternary ammonium compounds from the cells are responsible for a significant decrease in bacterial susceptibility [22,23,24]. In fact, the use of quaternary ammonium compounds has been linked to non-tuberculous mycobacteria infections following surgical procedures [25,26].

In a study conducted in Japan, no relation between multi-drug resistance and disinfectant-susceptibility in *M. tuberculosis* strains was found [27]. Sanitisers and disinfectants, contrarily to antimicrobials, interact non-specifically and, generally, on multiple cellular targets such as membranes, proteins, or genetic material which should reduce the probability of bacteria becoming resistant to these compounds [28]. Nevertheless, alcohol-resistant/tolerant bacteria, including mycobacteria strains, have been isolated [29,30]. The aim of this study is thus to study *M. vaccae* adaptation to disinfectants used in health care facilities, and to determine if the adapted cells present higher tolerance to antibiotics and EPIs than their non-adapted counterparts. This should be considered before using *M. vaccae* cells as a novel therapeutic agent.

## 2. Results

### 2.1. Membrane Fatty Acid Composition Induced by Disinfectants

**Figure 1:**
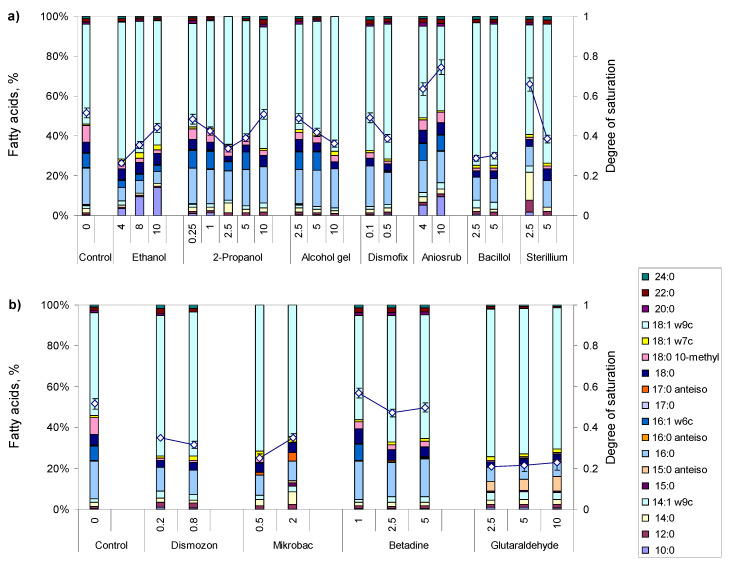
Fatty acid composition, and respective calculated degree of saturation, of *M. vaccae* cells grown in the absence (Control in both a and b) and in the presence of either alcohol (**a**) or non-alcohol (**b**) based disinfectants. Only FA accounting for at least 1% of total lipids are represented. The content of each fatty acid presented here may be found in Table S1.

To evaluate the effect of disinfectants commonly used in health care facilities on *M. vaccae* cells, the disinfectants were initially added at sub-lethal concentrations to cultures in mid-exponential phase. This classical approach, to study the effect of toxic compounds, allows de novo synthesis of fatty acids, if necessary, for the response of the cells at the membrane composition level [19,31]. After the addition of the disinfectant, the cells were monitored for 8 h. For comparison, the fatty acid (FA) composition of the exposed *M. vaccae* cells is presented after 6 h, which is higher than one duplication time in the presence of all tested disinfectants (Figure 1, Supplementary Table S1).

Both alcohol (Figure 1a) and non-alcohol (Figure 1b) based disinfectants induced significant dose-dependent changes in the FA profile of *M. vaccae* cells, when compared to unchallenged control cells. Ethanol, Aniosrub 85 NPC and Bacillol^®^ caused a dose dependent increase in the degree of saturation of the FAs, whilst the remaining alcohol-based disinfectants caused a decrease (Figure 1a). This cell behaviour is contrary to what was observed with *Rhodococcus erythropolis* cells, which belong to the same Corynebacteriaceae family: they responded to short-chain alcohols, such as ethanol, with a concentration-dependent decrease in the membrane degree of saturation [32]. The increase in the degree of saturation in *M. vaccae* cells, in the case of ethanol, was mainly the result of a 3.7-fold increase in the amount of the saturated FA 10:0 and a concomitant 0.8-fold decrease in the amount of the mono-unsaturated FA 18:1ω9c. However, the values observed for the degree of saturation in the presence of ethanol and Bacillol were lower than that observed in unchallenged cells, but cells exposed to Aniosrub presented a degree of saturation 1.2 to 1.4-fold higher than control cells (Figure 1a). Cells exposed to Betadine^®^, an iodine based antimicrobial solution, presented a dose dependent decrease in the degree of saturation but, up to a concentration of 1% (*v*/*v*), the value was similar to that shown by control cells (Figure 1b). *M. vaccae* cells grown in the presence of Dismozon^®^ plus (an oxygen-active surface disinfectant), Mikrobac^®^ (an aldehyde-free surface disinfectant), and glutaraldehyde presented a degree of saturation that was, on average, 64%, 58%, and 42% that of unchallenged cells. Curiously, the cells decreased the degree of saturation up to a concentration of 2.5% of 2-propanol, but increased the saturation degree for increasing concentrations of this alcohol.

The results suggest that the cells responded to the tested surfactants by increasing the fluidity of the cellular membrane. Additionally, with exception of alcohol gel, Dismofix^®^ G and Aniosrub, cells increased significantly the amount of *anteiso* branched FA (Figure 1). These FA contain the methyl group at the antepenultimate carbon atom from the end of the FA, and present lower phase transition temperatures than their *iso*-counterparts [33,34], thus further contributing to an increased fluidity of the membrane of the cells.

### 2.2. Effect of the Culture Age during Exposure

In health care facilities where the disinfectants are used, bacteria are hardly growing on the surfaces to be disinfected. They should be in a viable non-growing state, and to survive they should be able to make the necessary adjustments in the FA membrane composition without *de novo* synthesis of FA. To assess this hypothesis, *M. vaccae* cells were exposed both during the exponential and stationary phases to disinfectants Dismozon, Dismofix and Aniosrub.

**Figure 2:**
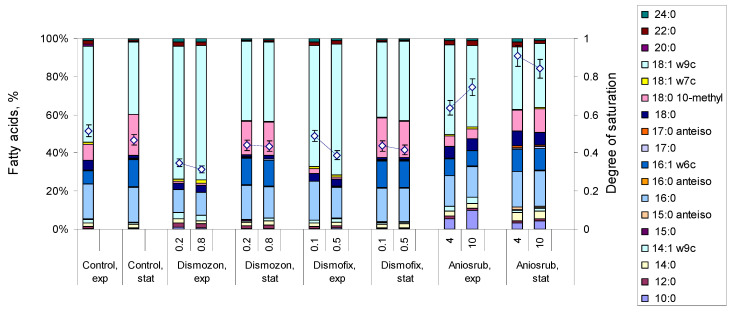
Fatty acid composition of *M. vaccae* cells exposed for 6h to disinfectants during exponential (exp) and stationary (stat) growth phases. Only FA accounting for at least 1% of total lipids are represented. The content of each fatty acid presented here may be found in Table S2.

Control *M. vaccae* cells, not previously exposed to disinfectants, presented lipid profiles according to the growth phase. A 2.6- and 2.0-fold increase in, respectively, the amount of 10-methyl 18:0 and 16:1 ω6c, and a 0.8-fold decrease in the amount of 18:1 ω9c were observed in cells collected during the stationary phase when compared to those harvested during the exponential phase (Figure 2, Supplementary Table S2). When comparing control cells to those exposed to disinfectants, it is clear that cells exposed during the stationary phase presented FA composition similar to unchallenged stationary phase control cells with the same age. On the contrary, cells exposed to disinfectants during the exponential phase could change considerably their FA profile. In the presence of the surface cleaners Dismozon and Dismofix, exponentially growing *M. vaccae* cells decreased on average 6.9- and 4.1-fold, respectively, the amount of 18:0 10-methyl, when compared to control cells, and stopped producing 16:1 ω6c. Concomitantly, they increased 1.4- and 1.3-fold the content of 18:1 ω9c in the presence of Dismozon and Dismofix, respectively. In the case of Aniosrub, the major adaptation in the lipid profile of exponentially growing cells was a dose dependent increase (up to 25.6-fold with 10% Aniosrub) in the amount of the saturated straight 10:0 FA, and a 1.2-fold increase in the amount of 18:0 for both tested concentrations. In fact, cells exposed to Aniosrub during both the exponential and stationary phases presented a significantly higher degree of saturation than their corresponding control counterparts, indicating that they required a more rigid membrane to grow and survive in its presence. The results thus indicate that *M. vaccae* cells could make adjustments in the lipid composition, even during the stationary phase.

### 2.3. Adaptation of M. vaccae Cells to Disinfectants

**Figure 3:**
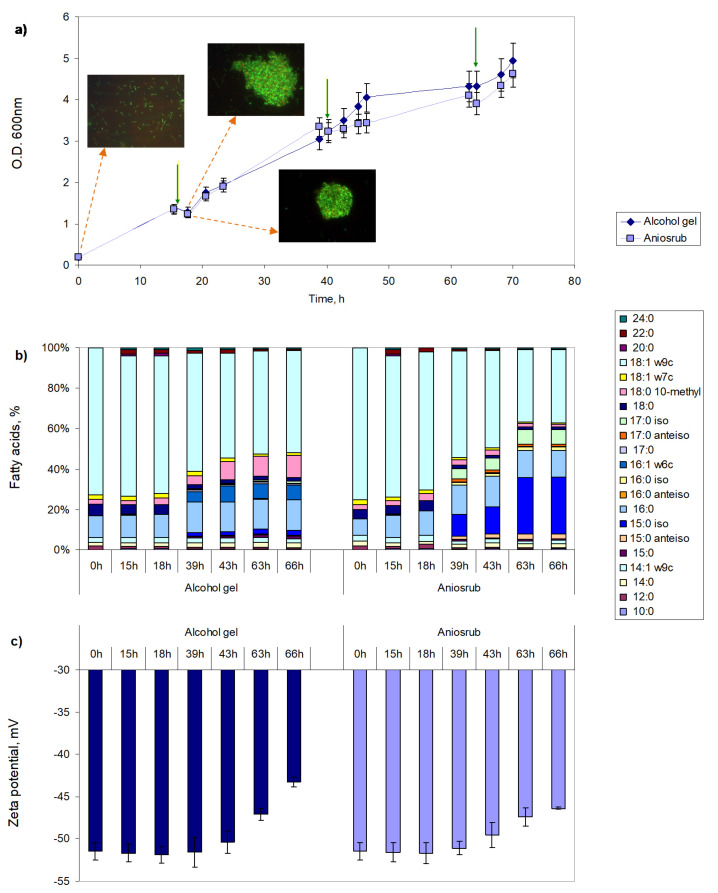
Adaptation of *M. vaccae* cells to alcohol gel and Aniosrub. (**a**) The disinfectants were added at 16, 40, and 64 h of growth (green arrows), causing cell aggregation in the following hour (fluorescence microscopy images indicated by orange arrows), which decreased the optical density (O.D. 600 nm) before growth was resumed. (**b**) Fatty acid composition of the cells at time zero, one hour prior disinfectant addition and at least two hours following addition. (**c**) Zeta potential of the cells measured at the same time as in (**b**). Only FA accounting for at least 1% of total lipids are represented. The content of each fatty acid presented in (**b**) may be found in Table S3.

A key aspect of the wide use of disinfectants is to know if bacteria exposed to them may adapt and become tolerant/resistant to other compounds such as antibiotics. To assess that, *M. vaccae* cells were adapted to alcohol gel, used as hand sanitiser, and to Aniosrub, a hand-rubbing solution used for hygienic treatment and surgical disinfection. The cells could use both disinfectants as sole carbon source at concentrations of 0.25% and 1% (*v*/*v*) (data not shown). The stepwise strategy to adapt the cells to the disinfectants was to grow the cells in Mueller-Hinton broth and to add each disinfectant solution to reach a concentration of 2.5% (*v*/*v*) during the exponential phase at 16, 40 and 64 h (Figure 3a). To assess the changes, the cell lipid composition and zeta potential were studied before and after the addition of the disinfectant (Figure 3b,c; Table S3).

After the several additions of both alcohol gel and Aniosrub, cell aggregates were formed, which resulted in the decrease of the optical density (O.D. 600 nm) values in the immediate following hours of growth (Figure 3a). Nevertheless, cells could grow for at least 70 h. After 39 h of cultivation, and especially following the second addition of each disinfectant, cells changed their FA composition (Figure 3b). Following two alcohol gel additions, cells increased 3.6-fold the content of 10-methyl 18:0 and started producing 16:1 ω6c (reaching ca. 7.5% of total FAs at 43 h), while decreasing 1.4-fold the content of 18:1 ω9c, when compared to cells after 15 h of growth. During adaptation to Aniosrub, *M. vaccae* increased the content of 16:0 from 8 to 12%, while decreasing 3.9-fold the amount of 18:0 and 2.2-fold the content of 18:1 ω9c (Figure 3b; Supplementary Table S3). However, the most curious adaptation was the production of large amounts of *iso*-branched FAs, in particular 15:0 *iso* and 17:0 *iso*, which had not been previously observed. Both 15:0 *iso* and 17:0 *iso* FAs were first observed at 39 h of cultivation and the cells steadily increased their production which reached, respectively, 27 and 7% of total FAs at 66 h. The amount of 16:0 *iso* reached only 1.6%. The amount of these FAs was lower when the cells were adapted to alcohol gel, with 15:0 *iso* reaching 2.6% of total FAs, but they were also observed at 39 h of cultivation.

Regarding the net surface charge of the cells, a decrease in zeta potential towards less negative values was observed following the second addition of both alcohol gel and Aniosrub (Figure 3c). Unchallenged cells presented a zeta potential of −51.5 ± 1.0 mV whilst at 66 h, following the third addition of alcohol gel and Aniosrub, the cells showed zeta potential values of −43.3 ± 0.6 mV and −46.4 ± 0.1 mV, respectively. The alcohol gel presents a zeta potential of −17.7 ± 0.3 mV and Aniosrub a value of −8.3 ± 0.8 mV (data not shown). Since the decrease in the zeta potential of the cells was only significant after the second disinfectant addition, this could indicate that both alcohol gel and Aniosrub were accumulating at the cellular membrane. The major compound of both disinfectants is ethanol which is known to only penetrate slightly into the hydrophobic area of membranes, remaining in the hydrophilic headgroup area of the phospholipids [32,35,36].

### 2.4. Tolerance of Disinfectant Adapted Cells to Antibiotics and Efflux Pump Inhibitors

To assess if *M. vaccae* cells adapted to disinfectants are more tolerant to the antibiotics levofloxacin and teicoplanin, the minimum inhibition concentration (MIC) for each antibiotic was determined for both non-adapted and disinfectant-adapted cells by the broth microdilution method. The same method was used to determine if disinfectant-adapted cells were more tolerant towards the EPIs thioridazine and omeprazole. Adapted cells were more tolerant to levofloxacin, thioridazine, and omeprazole, than non-adapted cells (Table 1). A 2-fold increase in the MIC of levofloxacin towards cells adapted to alcohol gel and an eight-fold increase in the MIC towards cells adapted to Aniosrub was observed, when compared to non-adapted counterparts. The MIC of omeprazole also doubled in disinfectant adapted cells, and increased at least eight-fold for thioridazine, when compared to control cells. The cells could grow in the presence of at least 100 µg/mL teicoplanin.

**Figure 4:**
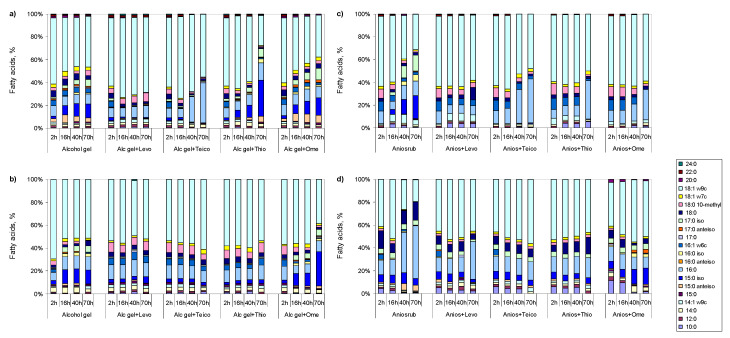
Fatty acid composition of non-adapted cells of *M. vaccae* (**a**,**c**) and previously adapted cells to alcohol gel (**b**), and Aniosrub (**d**), when exposed to the disinfectants (alcohol gel, a,b; Aniosrub, c,d), antibiotics, and efflux pump inhibitors. The antibiotics tested were levofloxacine (Levo), teicoplanin (Teico), and the efflux pump inhibitors were thioridazine (Thio), and omeprazole (Ome). Only FA accounting for at least 1% of total lipids are represented. The content of each fatty acid presented here may be found in Table S4.

*M. vaccae* cells, both non-adapted and adapted to disinfectants, made substantial phenotypic modifications at the lipid level when exposed to half the MIC of each antibiotic and EPIs (Figure 4; Supplementary Table S4). The cells were initially cultivated without and with adaptation to Aniosrub and alcohol gel, as described previously, and were used to inoculate new cultures containing half the MIC of disinfectants and half the MIC of levofloxacin, teicoplanin, thioridazine, or omeprazole.

Cells that were not adapted responded to alcohol gel, and to this disinfectant plus thioridazine and omeprazole, mainly with a dose dependent increase in the amount of 15:0 *iso* and 17:0 *iso*, while decreasing the amount of 18:1 ω9c (Figure 4a). In the presence of alcohol gel and teicoplanin, the cells adopted a more rigid membrane by increasing the amount of 16:0 and 18:0, whilst during exposure to levofloxacin, the cells produced much fewer changes. Similarly, cells not adapted when exposed to Aniosrub and teicoplanin or EPIs increased 2–3-fold the amount of 16:0 between 2 and 70 h, while decreasing or maintaining the content of 16:1 ω6c and 18:1 ω9c (Figure 4c). When the cells were exposed to Aniosrub alone, they contained up to 18% of 15:0 *iso* and 12% of 17:0 *iso* at 70 h of culture.

*M. vaccae* cells that were adapted to alcohol gel and Aniosrub presented less changes throughout time when exposed to antibiotics and EPIs (Figure 4b,d) than non-adapted cells (Figure 4a,c). Nevertheless, cells adapted to alcohol gel presented significant amounts of *iso*-branched FAs, which represented up to 21.9% of total FA content at 70h (Figure 4c). Cells adapted to Aniosrub presented up to 9.4% of *iso*-branched FAs at 70 h of growth when exposed to antibiotics and EPIs (Figure 4d). However, cells adapted to Aniosrub and exposed to antibiotics decreased two-fold the content of the long saturated 18:0. The content of 16:0 increased 3-fold in cells adapted to Aniosrub and further exposed to the disinfectant, and increased 2.2- and 1.2-fold when these cells were exposed to levofloxacin and teicoplanin, respectively.

Aggregation of cells into large clusters occurred during adaptation to both disinfectants (Figure 3a). At the end of the adaptation period, fluorescence microscopy observations showed that the cells produced an exopolymeric substance which surrounded the cell aggregates (data not shown). Such production stopped, apparently, when the cells were exposed to teicoplainin. In this case, the cells also decreased significantly their size.

## 3. Discussion

In recent years, *M. vaccae* has been proposed in unconventional treatments due to its immunoregulatory and anti-inflammatory properties. Among the therapies proposed are those for the treatment of trauma- and stress-related psychiatric disorders [37,38,39], to increase long-term survival and outcome of patients with metastatic malignant melanoma [10] and non-small-cell lung cancer [9], and as adjuvant to antituberculosis chemotherapy [40,41]. *M. vaccae*, like other non-tuberculous mycobacteria, is generally regarded as non-pathogenic, although it has been implicated in pulmonary and skin infections [6].

Nontuberculous *Mycobacterium* species are often abundant in microbial communities in, e.g., soil, lakes, rivers, and water distribution systems [42,43]. Their environmental tenacity has been linked to a remarkable solute-stress tolerance [44], but their hydrophobic cell envelope acting as a permeability barrier is probably the most important contributor to the ecology and epidemiology of mycobacteria [45]. The remarkable impermeability of the mycobacterial cell wall results from the mycolic acids, as well as the cross-linked glycan strands of the cell envelope, being arranged predominantly perpendicularly to the cell wall surface [46,47]. Nevertheless, a model containing a lipid bilayer as outer membrane and an inner membrane, rich in diacyl phosphatidylinositol dimannosides, which also contribute to the generalized drug resistance of mycobacteria, has been proposed [48]. We have also shown that changes in the lipid composition of the cell envelop of *M. vaccae* contribute to the adaptation of this bacterium to organic solvents [19].

Bacterial resistance to antibiotics is a global health problem which has been attributed to overuse and misuse of these compounds in humans and in animal farms (e.g., [49,50]). Cross-resistance and co-resistance between disinfectants and antibiotics is much less studied but it has been shown that they occur in the case of quaternary ammonium compounds [22], and with triclosan, octenidine, sodium hypochlorite, and didecyldimethylammonium chloride [51]. However, little is known from increased tolerance resulting from phenotypic adaptations of the cells exposed to disinfectants and antiseptics. Modifications to, e.g., the FA composition of the cellular membrane of bacterial cells, may hamper the entrance of other compounds, such as antibiotics and disinfectants, in the cell and prevent their action, or cells may convert the compound into non-toxic forms [2,19]. Such temporary, phenotypic modification may help the cells to tolerate the action of antibiotics and efflux pump inhibitors. Furthermore, phenotypic adaptations have been found to be transmissible to the following generation [52]. To test our hypothesis, we selected two antibiotics from classes active against mycobacteria and two EPIs. These latter compounds have been used in multi-drug TB therapies to restore drug susceptibility through efflux pump inhibition which results in increased intracellular drug concentration.

The results of the present study showed that *M. vaccae* cells were able to make phenotypic adjustments to grow in the presence of several alcohol and non-alcohol-based disinfectants. Ethanol, Aniosrub, and Bacillol caused a dose dependent increase in the degree of saturation of the FA, whilst the other disinfectants caused the opposite response (Figure 1). Cells grown in the presence of Aniosrub presented a degree of saturation 1.2 to 1.4-fold higher than control cells. Furthermore, cells exposed to this hand sanitiser showed higher degree of saturation than their corresponding control counterparts. However, to other tested disinfectants and hand sanitisers, the cells responded by decreasing the degree of saturation of the FAs of their cellular membrane and by changing the content of branched saturated FA. During growth, the cells could thus make the necessary adjustments to survive and thrive in the presence of the tested solutions.

When *M. vaccae* cells were exposed to the disinfectants during the stationary growth phase, their composition was similar to control cells, which could be the result of an inability to change FA composition by de novo synthesis. However, cells exposed to Aniosrub during the stationary phase presented a degree of saturation that nearly doubled that presented by unexposed control cells. This was mainly the result of an increase in the content of the saturated straight 10:0, 14:0, and 18:0 FAs, and a concomitant decrease in the amount of the monosaturated 16:1 ω6c and 18:1 ω9c FAs (Figure 2). This suggests that *M. vaccae* cells might be able to convert monounsaturated into saturated FA by an enzyme driven mechanism not requiring *de novo* synthesis. In *M. tuberculosis*, enzymes such as DesA3, a membrane-bound stearoyl-CoA Δ9 desaturase, have been found able to carry out the desaturation of 16:0 and 18:0 to 16:1 and 18:1, respectively, but they also showed some saturation properties. We have also previously shown that *R. erythropolis* is able to respond to salt stress rapidly, in few minutes, by making significant changes in the FA composition of the cells, including the production of polyunsaturated FA [53]. The ability of making rapid changes in their membrane, when exposed to environmental stress, could be one of the strategies of *Mycobacterium* and *Rhodococcus* species to survive in a myriad of environmental conditions and in the presence of deleterious compounds.

When adaptation to alcohol gel and Aniosrub was promoted by making three disinfectant additions during *M. vaccae* growth, significant changes in the lipid profile of the cells was observed mainly following the second addition (Figure 3). In the case of adaptation to gel alcohol, the cells increased the content of 10-methyl 18:0 and started to produce 16:1 ω6c, and decreased the amount of 18:1 ω9c. Methyl branching in FA reduces lipid condensation, chain ordering and lipid bilayer thickness, and enhances fluidity of the cellular membrane through the formation of kinks at the branching point [54]. *M. vaccae* cells adapted to Aniosrub presented increased content of 16:0 and decreased content of 18:0 and 18:1 ω9c, when compared to cells before adaptation. However, the most curious adaptation at the lipid level was the production of large amounts of *iso*-branched FA, in particular 15:0 *iso* and 17:0 *iso*. The *iso*-branched FAs present the branched methyl group at the penultimate carbon atom, whilst in the *anteiso* form, the methyl group is located at the antepenultimate carbon from the end. The melting temperatures of a saturated straight FA and the *iso*-FA with the same number of carbons are similar, but the phase transition temperature of the *iso*-acyl is lower, and that of the *anteiso* is even lower. The 15:0 *iso* has a transition temperature of 52.2 °C while the 15:0 *anteiso* presents a value of 25.8 °C [33,34]. At phase transition temperature, half of the hydrocarbon chains of the FA melt and both a rigid gel phase and a liquid crystalline phase may be observed. As mentioned, the cells adapted to Aniosrub increased the amount of *iso*-FA and 16:0, and decreased the content of *anteiso*-FA, 18:0, and 18:1 ω9c. This indicates that the cells decreased the fluidity of the cellular membrane while adapting to Aniosrub. *M. vaccae* cells thus adjusted the transition temperature of phospholipids to achieve the best membrane permeability by making changes in the FA composition according to the disinfectant used. Aniosrub is composed by 755 mL/L ethanol whilst the alcohol gel tested contains, besides denaturated alcohol, other compounds such as 1-propanol, 2-propanol, glyceryl oleate, and PEG-7 glyceryl cocoate. The different compositions induced different changes in the *M. vaccae* cells: an increased fluidity as response to alcohol gel but a reduced fluidity in the presence of Aniosrub.

The zeta potential of the cells during adaptation to both alcohol gel and Aniosrub decreased with time and number of disinfectant additions (Figure 3c), indicating that alcohol could be accumulating at the hydrophilic headgroup area of the phospholipids since it can only penetrate slightly into the hydrophobic phospholipid bilayer. This causes a swelling effect on the hydrophilic headgroups [35]. Usually bacterial cells, such as *R. erythropolis* and *Acinetobacter calcoaceticus*, respond to short-chain alcohols by decreasing the degree of saturation of the FAs of the membrane [32,36]. However, *M. vaccae* cells increased the degree of saturation with increasing concentrations of ethanol, 2-propanol, and Aniosrub (Figure 1 and Figure 3b). In addition to changes in the lipid composition and net surface charge, adapted cells also aggregated into large clusters and produced exopolymeric substances. This behaviour may have a strong impact in the entrance of compounds into the cells and could influence their tolerance towards other compounds. To test this, the disinfectant adapted cells were exposed to two antibiotics and two EPIs.

*M. vaccae* cells, that were not previously adapted, had to make substantial adjustments in lipid composition in response to the antibiotics teicoplanin and levofloxacin, and to the EPIs thioridazine and omeprazole. In general, non-adapted cells decreased the fluidity of the membrane when exposed to these compounds. Adapted cells did not require as extensive changes in the lipid composition as those not adapted to grow in the presence of half the MIC of the tested antibiotics and EPIs. However, those adapted still increased significantly the content of *iso*-branched FA, reaching ca. 22% if previously adapted to alcohol gel, and ca. 10% if adapted to Aniosrub.

In a previous study, we showed that *M. vaccae* cells adapted to ethanol and methyl *tert*-butyl ether increased the tolerance towards thioridazine and omeprazole but became more susceptible to levofloxacin and teicoplanin [19]. In the present study, cells adapted to disinfectants increased their tolerance towards levofloxacin: two-fold in the case of alcohol gel adapted cells and eight-fold in the case of cells adapted to the hand disinfectant Aniosrub, when compared to non-adapted cells (Table 1). In adapted cells, the MIC of omeprazole doubled and increased at least 8-fold for thioridazine. In the case of teicoplanin, the cells grew in the presence of at least 100 µg/mL, regardless of being adapted or not. Teicoplanin is a glycopeptide that acts by interacting with the d-Ala-d-Ala terminus of the pentapeptide side chain of peptidoglycan precursors, thus preventing polymerization reactions [55]. The antibiotic is secreted by *Actinoplanes teichmyceticus* as a mixture of congeners with different fatty-acid chains. Since the molecule is hydrophobic because the acyl substitute of the *N*-acylglucosamine is a FA containing 10–11 carbon atoms, teicoplanin is able to cross membranes. Teicoplanin is thus a good model antibiotic to assess if changes in the lipid profile of the cells affect the entrance of antimicrobials into the cells. Additionally, it has also been used to treat infections caused by fast growing non-tuberculous mycobacteria making this assessment relevant for future applications. In this study, the changes in fatty acid profile of the cells prevented teicoplanin entrance into the cells resulting in increased MIC value, but when the cells were adapted to organic solvents, the cells became susceptible to this antibiotic [19]. This indicates that modulation of the permeability of the cells influences the entrance of antimicrobials in *M. vaccae*. The work of Keiser et al., with mutants presenting different susceptibility to drugs affecting cell-wall components synthesis but not the detergent sodium dodecyl sulfate, suggests that cell-wall synthesis and not permeability is responsible for teicoplanin susceptibility [19,56]. Nevertheless, the impermeability of the mycobacterial cells wall, the action of efflux pumps, the enzymatic modification of antibiotics, and the modulation of gene expression, have been found to be important physiological responses, in addition to target mutations, for intrinsic and acquired drug resistance and tolerance mechanisms in mycobacteria [57]. In the case of the fluoroquinolone levofloxacin, which both zwitterionic and neutral forms cross membranes via passive transport, the modifications the cells underwent during adaptation to disinfectants were sufficient to prevent it from entering the cells. The changes in the FA composition should have increased the impermeability of the cell envelop of *M. vaccae* towards levofloxacin, but changes in the net surface charge should also have contributed to prevent the entrance of the zwitterionic form resulting in increased MIC values.

The use of an EPI in combination with antibiotics can prevent efflux pump related antibiotic resistance and/or enhance the activity of certain antibiotics. The EPI thioridazine has been shown to alter the permeability of the cell envelop of *M. tuberculosis* and to interact with the bacterial plasma membrane increasing also its permeability [58]. Omeprazole, a gastric acid suppressive agent widely used to treat acid reflux and ulcers, has been associated to TB infection/activation [59]. Since *M. vaccae* is being tested for use as an adjuvant therapy to anti-tuberculosis chemotherapy, it is important to assess the effect of disinfectant adaptation to omeprazole tolerance on *M. vaccae*. The modifications produced by the cells during adaptation resulted in a two-fold increased MIC of omeprazole (Table 1). EPIs should be able to pump both antibiotics and organic solvents such as those presented in disinfectants and hand sanitisers [2,60]. Therefore, during adaptation, not only modifications at the lipid level, but also in the number and/or activity of efflux pumps per cell may have occurred.

In a year marked by the COVID-19 pandemic, which led to the widespread use of disinfectants and hand rubbing solutions, further studies are necessary to assess if bacterial cells that survive in human skin and surfaces following disinfection, will present increased tolerance/resistance to, e.g., antibiotics. Our study strongly suggests that continuous exposure to disinfectants and sanitisers contributes to increased mycobacterial tolerance to antibiotics and EPIs.

## 4. Materials and Methods

### 4.1. Bacterial Strain and Growth Conditions

*Mycobacterium vaccae* ATCC 15483 was grown in 100 mL Erlenmeyer flasks containing 20 mL of Mueller-Hinton broth (purchased from Sigma-Aldrich, St. Louis, MO, USA) supplemented with 0.1% Tween 80 (from Merck-Schuchardt, Hohenbrunn, Germany), at 30 °C and 200 rpm in an incubator Agitorb 200 (Aralab, Rio de Mouro, Portugal). Cell growth was monitored by optical density (OD) measurements at 600 nm, using a Spectrophotometer U2000 (Hitachi, Tokyo, Japan).

### 4.2. Bacterial Growth in the Presence of Disinfectants

Each disinfectant was added, at a certain concentration, to mid-exponential phase cultures in Mueller-Hinton broth, with an OD at 600 nm of 1.0 ± 0.2. Growth was monitored and maintained under the conditions mentioned in Section 4.1. The disinfectants and hand sanitisers tested were the following: ethanol (>99.9%, from Panreac, Barcelona, Spain); 2-propanol (99.9%, Sigma-Aldrich); alcohol gel (Cutan^®^ Gel Hand Sanitiser from Deb, Denby, UK); glutaraldehyde (50% in water, from Sigma-Aldrich); Dismofix^®^ G, Bacillol^®^ 30 Foam, Sterillium^®^, Dismozon^®^ plus, Mikrobac^®^ forte (all from Bode Chemie GmbH, Hamburg, Germany); Betadine^®^ (from Meda Pharma, Lisboa, Portugal); and Aniosrub 85 NPC (from Laboratoires Anios, Lezennes, France).

To test if the *M. vaccae* cells could use the disinfectants as carbon source, the cells were grown in 100 mL Erlenmeyer flasks containing 20 mL of mineral salts medium described previously [61], and 0.25% or 1% (*v*/*v*) of disinfectant. Cultures were grown at 30 °C and 200 rpm and growth was monitored by OD measurements at 600 nm, as previously mentioned.

Assays were done at least in duplicate.

### 4.3. Bacterial Adaptation to Disinfectants and Hand Sanitisers

*M. vaccae* cells were adapted to alcohol gel and Aniosrub by using a stepwise strategy previously used by de Carvalho et al. [62]. Briefly, pulses of each disinfectant to reach a concentration of 2.5% (*v*/*v*) were added to 40 mL cell cultures growing in Mueller-Hinton media supplemented with 0.1% Tween 80, once the culture reached mid-exponential phase. Further additions were made to the cultures whenever they reached exponential growth again. The growth was monitored and conditions kept as previously described. The assays were carried out in duplicate.

### 4.4. Fatty Acid Composition

To assess the changes in FA composition of the cells induced by each disinfectant and hand sanitiser, 1 mL samples of cell suspension were collected from *M. vaccae* cultures at the time mentioned in each section. During adaptation, samples were collected before and during exposure to the tested compounds. After harvesting, samples were centrifuged at 10,000 rpm for 5 min, and the pellet was washed twice with milli-Q water. The FAs of the cells were extracted and simultaneously methylated to fatty acid methyl esters (FAMEs), using the instant-FAME method from MIDI, Inc. (Newark, DE, USA) The analysis of FAMEs was carried out in a 6890N gas chromatograph from Agilent Technologies (Palo Alto, CA, USA), equipped with a flame ionization detector and an automatic injector 7683B. A 25 m long Agilent J&W Ultra 2 capillary column was used to separate the FAMEs. Each FAME was identified by the PLFAD1 method of Sherlock^®^ software version 6.2 from MIDI, Inc. The saturation degree was defined as the ratio between the sum of the percentage of saturated FAs and the sum of the percentage of monounsaturated FAs present in the cells.

### 4.5. Zeta Potential Measurements

To determine the zeta potential of *M. vaccae*, 1 mL of cell suspension was collected before and during exposure to disinfectants and hand sanitisers. The collected samples were centrifuged at 10,000 rpm for 5 min and washed at least three times with milli-Q water. The zeta potential of the bacterial cells, resuspended in 10 mM KNO_3_ at pH 6.2, was determined by a Doppler electrophoretic light scattering analyzer (Zetasizer Nano ZS, from Malvern Instruments Ltd., Malvern, UK). The zeta potential was determined from the electrophoretic mobility of the cells at 25 °C, according to the method of Helmholtz–von Smoluchowski, by the Zeta Software version 7.11 (Malvern Instruments Ltd.). The zeta potential of the disinfectants was determined using a Glass “Dip” cell (also from Malvern Instruments Ltd.).

### 4.6. Fluorescence Microscopy

Cell viability and morphology were observed and determined using a LIVE/DEAD^®^ *Bac*Light^TM^ Bacterial Viability Kit from Molecular Probes (Invitrogen, Thermo Fisher Scientific, Waltham, MA USA). Observations were made on an Olympus CX40 microscope (from Olympus, Tokyo, Japan) equipped with an Olympus U-RFL-T burner and a U-MWB mirror cube unit (excitation filter: BP450–480; barrier filter: BA515). Images were collected with an Evolution^TM^ MP 5.1 CCD color camera using the acquisition software Image-Pro Plus (both from Media Cybernetics, Rockville, MD, USA). Cell viability and morphological features were calculated by image analysis using Visilog 5 (Noesis SA, Crolles, France) as previously described [63].

### 4.7. Determination of Minimum Inhibitory Concentration

The minimum inhibitory concentration (MIC) of the antibiotics teicoplanin and levofloxacin, and of the EPIs thioridazine and omeprazole (all from Sigma-Aldrich), towards *M. vaccae* was determined by the broth microdilution method according to CLSI guidelines [64]. In summary, antibiotics and EPIs were serially diluted in two-fold steps, to a volume of 150 µL of Mueller-Hinton broth. The initial concentrations of the antibiotics and EPIs were the following: 10 and 7.5 µL/mL of levofloxacin; 100 and 75 µL/mL of teicoplanin; 149 and 125 µL/mL of thioridazine, and 500 and 400 µL/mL of omeprazole. To each well of a 96-well plate, containing the 150 µL of broth with the compounds to be tested, 50 µL of cell suspension, collected in the exponential phase and diluted in Mueller-Hinton broth to reach a 0.5 McFarland standard, was added. Both non-adapted and cells adapted to alcohol gel and Aniosrub were used. The 96-well plates were covered with Breathe-Easy^TM^ sealing membranes (from Sigma-Aldrich) and incubated at 30 °C. The MICs were determined by measurement of the OD at 600 nm after ca. 72 h of growth on a spectrophotometer SpectraMax^®^ Plus 384 from Molecular Devices (San Jose, CA, USA). The assays were carried out in duplicate.

## Figures and Tables

**Table 1:** Minimum inhibitory concentrations (MIC) of the antibiotics levofloxacin and teicoplanin, and of the efflux pump inhibitors thioridazine and omeprazole, toward *M. vaccae* cells not adapted and adapted to the disinfectants alcohol gel and Aniosrub.

Antibiotic/EPI	MIC (µg/mL)		
	Non-Adapted Cells	Adapted Cells	
		Alcohol Gel	Aniosrub
Levofloxacin	0.6	1.25	5
Teicoplanin	>100	>100	>100
Thioridazine	18.7	149	>149
Omeprazole	250	500	500

## Supplementary Materials

The following are available online at https://www.mdpi.com/2079-6382/9/9/544/s1, Table S1: Fatty acid composition, and respective calculated degree of saturation, of *M. vaccae* cells grown in the absence and in the presence of disinfectants.; Table S2: Fatty acid composition of *M. vaccae* cells exposed for 6 h to disinfectants during exponential (exp) and stationary (stat) growth phases; Table S3: Fatty acid composition of the cells at time zero, one hour prior disinfectant addition and at least two hours following addition; Table S4: Fatty acid composition of *M. vaccae* of non-adapted cells and previously adapted cells to alcohol gel and Aniosrub, when exposed to the disinfectants, antibiotics, and efflux pump inhibitors.
