# Supplementary material for: Mycobacterium vaccae Adaptation to Disinfectants and Hand Sanitisers, and Evaluation of Cross-Tolerance with Antimicrobials
Source: Antibiotics (Basel). 2020 Aug 27;9(9):544. doi: 10.3390/antibiotics9090544 (PMC7559525; doi:10.3390/antibiotics9090544)
Supplement: Supplementary file 1 [file antibiotics-09-00544-s001.doc]

**Supplementary material**

**Table S1.** Fatty acid composition, and respective calculated degree of saturation (Deg Sat), of *M. vaccae* cells grown in the absence and in the presence of disinfectants.

| **Disinfectant** | **%, v/v** | **10:0** | **12:0** | **14:0** | **14:1 w9c** | **15:0** | **15:0**  ***anteiso*** | **16:0** | **16:0**  ***anteiso*** | **16:1 w6c** | **17:0** | **17:0**  ***anteiso*** | **18:0** | **18:0**  **10-methyl** | **18:1 w7c** | **18:1 w9c** | **20:0** | **22:0** | **24:0** |  | **Deg Sat** |
| --- | --- | --- | --- | --- | --- | --- | --- | --- | --- | --- | --- | --- | --- | --- | --- | --- | --- | --- | --- | --- | --- |
| Control | 0 | 0.37 | 1.00 | 2.00 | 1.64 | 0.19 | 0.50 | 17.87 | 0.18 | 6.90 | 0.28 | 0.19 | 5.05 | 8.34 | 0.84 | 49.49 | 1.00 | 1.67 | 1.09 |  | 0.52 |
| Ethanol | 4 | 3.27 | 0.72 | 1.01 | 2.01 | 0.00 | 0.00 | 6.03 | 0.00 | 3.55 | 0.29 | 0.00 | 5.13 | 2.18 | 2.40 | 64.63 | 0.56 | 1.28 | 0.89 |  | 0.26 |
|  | 8 | 8.28 | 0.66 | 1.01 | 0.00 | 0.00 | 0.00 | 5.58 | 0.00 | 2.98 | 0.25 | 0.00 | 5.00 | 1.97 | 2.29 | 58.94 | 0.42 | 0.94 | 0.69 |  | 0.36 |
|  | 10 | 12.16 | 0.73 | 1.00 | 0.00 | 0.00 | 0.00 | 5.38 | 0.00 | 2.77 | 0.22 | 0.00 | 4.76 | 1.78 | 2.07 | 54.16 | 0.39 | 0.89 | 0.65 |  | 0.44 |
| 2-Propanol | 0.25 | 1.07 | 1.15 | 1.84 | 1.51 | 0.20 | 0.00 | 17.79 | 0.22 | 8.72 | 0.28 | 0.16 | 4.95 | 5.37 | 1.15 | 51.54 | 1.08 | 1.50 | 0.76 |  | 0.49 |
|  | 1 | 1.24 | 1.22 | 1.70 | 1.61 | 0.22 | 0.00 | 17.11 | 0.22 | 8.83 | 0.27 | 0.17 | 3.96 | 5.81 | 1.18 | 53.60 | 0.71 | 0.92 | 0.50 |  | 0.43 |
|  | 2.5 | 0.00 | 1.31 | 5.28 | 1.36 | 0.00 | 0.00 | 15.70 | 0.00 | 4.78 | 0.29 | 0.00 | 2.66 | 5.13 | 0.94 | 67.28 | 0.00 | 0.00 | 0.00 |  | 0.34 |
|  | 5 | 0.00 | 1.28 | 1.82 | 1.65 | 0.00 | 0.00 | 18.32 | 0.00 | 8.75 | 0.00 | 0.00 | 3.53 | 3.46 | 0.94 | 58.06 | 0.00 | 1.27 | 0.93 |  | 0.39 |
|  | 10 | 0.00 | 1.59 | 2.24 | 2.42 | 0.00 | 0.00 | 17.70 | 0.00 | 0.00 | 0.00 | 0.00 | 5.70 | 2.24 | 1.08 | 59.97 | 1.28 | 2.30 | 1.46 |  | 0.51 |
| Alcohol gel | 2.5 | 0.51 | 1.31 | 2.06 | 1.51 | 0.30 | 0.20 | 17.30 | 0.00 | 8.59 | 0.48 | 0.00 | 5.90 | 3.46 | 1.46 | 53.17 | 1.18 | 1.60 | 0.99 |  | 0.49 |
|  | 5 | 0.43 | 1.09 | 1.66 | 1.25 | 0.00 | 0.00 | 18.22 | 0.00 | 9.24 | 0.37 | 0.00 | 4.11 | 3.71 | 1.58 | 55.83 | 0.64 | 1.13 | 0.74 |  | 0.42 |
|  | 10 | 0.00 | 1.16 | 1.47 | 1.21 | 0.00 | 0.00 | 19.62 | 0.00 | 0.00 | 0.00 | 0.00 | 3.50 | 3.17 | 2.32 | 67.56 | 0.00 | 0.00 | 0.00 |  | 0.36 |
| Dismofix | 0.1 | 0.44 | 0.89 | 1.91 | 1.44 | 0.00 | 0.00 | 20.04 | 0.00 | 0.00 | 0.00 | 0.00 | 3.83 | 2.66 | 0.94 | 62.10 | 1.04 | 2.08 | 1.62 |  | 0.49 |
|  | 0.5 | 0.77 | 0.91 | 2.04 | 1.74 | 0.26 | 0.00 | 15.82 | 0.00 | 0.00 | 0.39 | 0.30 | 3.44 | 1.36 | 1.09 | 67.20 | 0.75 | 1.80 | 1.14 |  | 0.39 |
| Aniosrub | 4 | 5.36 | 1.31 | 2.67 | 2.24 | 0.00 | 0.00 | 15.96 | 0.00 | 8.39 | 0.30 | 0.00 | 6.21 | 5.26 | 0.88 | 45.90 | 1.53 | 2.06 | 1.13 |  | 0.64 |
|  | 10 | 9.51 | 1.21 | 2.66 | 3.12 | 0.00 | 0.00 | 15.67 | 0.16 | 7.89 | 0.28 | 0.00 | 5.90 | 5.18 | 0.78 | 42.03 | 1.59 | 2.21 | 1.22 |  | 0.75 |
| Bacillol | 2.5 | 0.52 | 1.45 | 1.97 | 3.73 | 0.00 | 0.00 | 11.02 | 0.00 | 0.00 | 0.00 | 0.00 | 3.31 | 1.36 | 1.31 | 69.82 | 0.00 | 1.72 | 1.47 |  | 0.29 |
|  | 5 | 0.00 | 1.58 | 1.46 | 3.33 | 0.00 | 0.00 | 11.48 | 0.00 | 0.00 | 0.00 | 0.00 | 3.83 | 1.26 | 1.43 | 68.24 | 0.90 | 1.47 | 1.20 |  | 0.30 |
| Sterillium | 2.5 | 1.61 | 5.90 | 13.83 | 3.31 | 0.00 | 0.00 | 9.65 | 0.00 | 0.00 | 0.00 | 0.00 | 3.56 | 0.94 | 1.37 | 54.21 | 1.16 | 1.84 | 1.23 |  | 0.66 |
|  | 5 | 0.00 | 2.14 | 1.83 | 0.00 | 0.00 | 0.00 | 13.05 | 0.00 | 0.00 | 0.00 | 0.00 | 5.66 | 1.36 | 1.61 | 67.53 | 0.00 | 2.21 | 1.70 |  | 0.38 |
| Dismozon | 0.2 | 0.93 | 2.45 | 2.01 | 3.29 | 0.00 | 0.00 | 11.81 | 0.00 | 0.00 | 0.00 | 0.00 | 3.19 | 1.17 | 1.15 | 68.32 | 0.85 | 2.26 | 1.82 |  | 0.35 |
|  | 0.8 | 0.64 | 2.38 | 1.43 | 2.80 | 0.00 | 0.00 | 12.03 | 0.00 | 0.00 | 0.00 | 0.00 | 3.55 | 1.23 | 1.98 | 70.37 | 0.00 | 1.91 | 1.68 |  | 0.31 |
| Mikrobac | 0.5 | 0.00 | 1.54 | 3.05 | 2.11 | 0.00 | 0.00 | 9.78 | 0.00 | 0.00 | 0.00 | 1.43 | 4.63 | 2.17 | 3.56 | 70.68 | 0.00 | 0.00 | 0.00 |  | 0.25 |
|  | 2 | 0.00 | 2.00 | 5.04 | 2.55 | 1.31 | 0.91 | 8.02 | 0.00 | 0.00 | 0.00 | 3.34 | 4.21 | 0.00 | 2.52 | 53.90 | 0.00 | 0.00 | 0.00 |  | 0.35 |
| Betadine | 1 | 0.39 | 1.21 | 1.74 | 1.33 | 0.17 | 0.00 | 18.75 | 0.19 | 7.87 | 0.29 | 0.17 | 6.98 | 3.46 | 1.13 | 50.68 | 1.52 | 2.32 | 1.36 |  | 0.57 |
|  | 2.5 | 0.36 | 1.17 | 2.05 | 2.53 | 0.00 | 0.00 | 16.62 | 0.21 | 0.00 | 0.25 | 0.71 | 5.15 | 2.42 | 1.21 | 61.59 | 1.40 | 2.40 | 1.42 |  | 0.47 |
|  | 5 | 0.40 | 1.14 | 2.00 | 2.55 | 0.22 | 0.00 | 18.26 | 0.26 | 0.00 | 0.27 | 0.47 | 4.97 | 2.70 | 1.17 | 60.52 | 1.30 | 2.17 | 1.26 |  | 0.50 |
| Glutaraldehyde | 2.5 | 0.79 | 1.38 | 1.86 | 3.54 | 0.49 | 4.31 | 6.26 | 0.00 | 0.00 | 0.00 | 0.45 | 2.25 | 0.59 | 1.40 | 66.08 | 0.31 | 0.79 | 0.62 |  | 0.21 |
|  | 5 | 0.87 | 1.34 | 2.05 | 3.48 | 0.50 | 4.95 | 6.43 | 0.00 | 0.00 | 0.00 | 0.53 | 2.33 | 0.58 | 1.44 | 64.44 | 0.00 | 0.74 | 0.67 |  | 0.22 |
|  | 10 | 0.72 | 1.29 | 2.06 | 3.05 | 0.61 | 6.27 | 6.73 | 0.00 | 0.00 | 0.00 | 0.69 | 2.42 | 0.61 | 1.44 | 60.71 | 0.00 | 0.59 | 0.59 |  | 0.23 |

**Table S2. Fatty acid composition of *M. vaccae* cells exposed for 6h to disinfectants during exponential (exp) and stationary (stat) growth phases, and respective calculated degree of saturation (Deg Sat).**

| **Disinfectant** | **%, v/v** | **10:0** | **12:0** | **14:0** | **14:1w9c** | **15:0** | **15:0 *anteiso*** | **16:0** | **16:0 *anteiso*** | **16:1w6c** | **17:0** | **17:0 *anteiso*** | **18:0** | **18:0**  **10‑methyl** | **18:1w7c** | **18:1w9c** | **20:0** | **22:0** | **24:0** | **Deg Sat** |
| --- | --- | --- | --- | --- | --- | --- | --- | --- | --- | --- | --- | --- | --- | --- | --- | --- | --- | --- | --- | --- |
| Control, exp | | 0.37 | 1.00 | 2.00 | 1.64 | 0.19 | 0.00 | 17.87 | 0.00 | 6.90 | 0.28 | 0.00 | 5.05 | 8.34 | 0.84 | 49.49 | 1.00 | 1.67 | 1.09 | 0.52 |
| Control, stat | | 0.25 | 0.40 | 1.87 | 0.75 | 0.33 | 0.00 | 18.25 | 0.36 | 14.04 | 0.38 | 0.21 | 1.43 | 21.35 | 0.29 | 37.48 | 0.24 | 0.85 | 0.72 | 0.47 |
| Dismozon, exp | 0.2 | 0.93 | 2.45 | 2.01 | 3.29 | 0.00 | 0.00 | 11.81 | 0.00 | 0.00 | 0.00 | 0.00 | 3.19 | 1.17 | 1.15 | 68.32 | 0.00 | 2.26 | 1.82 | 0.34 |
|  | 0.8 | 0.64 | 2.38 | 1.43 | 2.80 | 0.00 | 0.00 | 12.03 | 0.00 | 0.00 | 0.00 | 0.00 | 3.55 | 1.23 | 1.98 | 70.37 | 0.00 | 1.91 | 1.68 | 0.31 |
| Dismozon, stat | 0.2 | 0.35 | 1.32 | 2.05 | 0.76 | 0.41 | 0.00 | 17.88 | 0.33 | 13.78 | 0.44 | 0.22 | 1.14 | 17.34 | 0.31 | 41.14 | 0.27 | 0.55 | 0.59 | 0.45 |
|  | 0.8 | 0.32 | 1.82 | 2.15 | 1.17 | 0.32 | 0.00 | 15.71 | 0.34 | 13.07 | 0.39 | 0.26 | 1.57 | 16.75 | 0.54 | 39.97 | 0.23 | 0.78 | 0.72 | 0.44 |
| Dismofix,  exp | 0.1 | 0.44 | 0.89 | 1.91 | 1.44 | 0.00 | 0.00 | 20.04 | 0.00 | 0.00 | 0.00 | 0.00 | 3.83 | 2.66 | 0.94 | 62.10 | 0.00 | 2.08 | 1.62 | 0.48 |
|  | 0.5 | 0.77 | 0.91 | 2.04 | 1.74 | 0.26 | 0.00 | 15.82 | 0.00 | 0.00 | 0.39 | 0.30 | 3.44 | 1.36 | 1.09 | 67.20 | 0.00 | 1.80 | 1.14 | 0.38 |
| Dismofix,  stat | 0.1 | 0.30 | 0.39 | 1.87 | 0.83 | 0.33 | 0.19 | 17.36 | 0.36 | 13.62 | 0.37 | 0.49 | 1.21 | 20.62 | 0.28 | 39.05 | 0.25 | 0.75 | 0.69 | 0.44 |
|  | 0.5 | 0.23 | 0.40 | 2.21 | 0.85 | 0.33 | 0.00 | 17.15 | 0.34 | 13.61 | 0.38 | 0.38 | 1.14 | 18.87 | 0.33 | 40.90 | 0.24 | 0.60 | 0.63 | 0.42 |
| Aniosrub,  exp | 4.0 | 5.36 | 1.31 | 2.67 | 2.24 | 0.00 | 0.00 | 15.96 | 0.00 | 8.39 | 0.30 | 0.00 | 6.21 | 5.26 | 0.88 | 45.90 | 0.00 | 2.06 | 1.13 | 0.61 |
|  | 10.0 | 9.51 | 1.21 | 2.66 | 3.12 | 0.00 | 0.00 | 15.67 | 0.16 | 7.89 | 0.28 | 0.00 | 5.90 | 5.18 | 0.78 | 42.03 | 0.00 | 2.21 | 1.22 | 0.72 |
| Aniosrub,  stat | 4.0 | 2.65 | 0.98 | 3.46 | 1.12 | 0.34 | 1.18 | 15.21 | 0.15 | 9.36 | 0.90 | 0.81 | 6.27 | 8.90 | 0.53 | 26.97 | 0.00 | 2.05 | 1.56 | 0.88 |
|  | 10.0 | 3.47 | 1.02 | 3.21 | 1.13 | 0.32 | 0.66 | 15.29 | 0.17 | 9.41 | 0.79 | 0.51 | 5.34 | 10.21 | 0.57 | 27.40 | 0.00 | 1.33 | 0.86 | 0.82 |

**Table S3.** Fatty acid composition of the cells at time zero, one hour prior disinfectant addition and at least two hours following addition, and respective calculated degree of saturation (Deg Sat).

| **Disinfectant** | | **10:0** | **12:0** | **14:0** | **14:1 w9c** | **15:0** | **15:0**  ***iso*** | **15:0**  ***anteiso*** | **16:0** | **16:0**  ***iso*** | **16:0**  ***anteiso*** | **16:1 w6c** | **17:0** | **17:0**  ***iso*** | **17:0**  ***anteiso*** | **18:0** | **18:0**  **10-methyl** | **18:1 w7c** | **18:1 w9c** | **20:0** | **22:0** | **24:0** | **Deg Sat** |
| --- | --- | --- | --- | --- | --- | --- | --- | --- | --- | --- | --- | --- | --- | --- | --- | --- | --- | --- | --- | --- | --- | --- | --- |
| Alcohol gel | 0h | 0.00 | 2.08 | 1.62 | 2.54 | 0.00 | 0.00 | 0.00 | 10.48 | 0.00 | 0.00 | 0.00 | 0.00 | 0.00 | 0.00 | 5.84 | 2.37 | 2.32 | 72.75 | 0.00 | 0.00 | 0.00 | 0.26 |
|  | 15h | 0.59 | 1.17 | 1.61 | 2.71 | 0.17 | 0.00 | 0.00 | 10.77 | 0.00 | 0.15 | 0.00 | 0.25 | 0.00 | 0.18 | 4.38 | 2.33 | 1.71 | 68.74 | 1.18 | 1.90 | 1.08 | 0.32 |
|  | 18h | 0.58 | 1.28 | 1.66 | 2.51 | 0.00 | 0.00 | 0.00 | 11.50 | 0.00 | 0.00 | 0.00 | 0.00 | 0.00 | 0.00 | 4.70 | 3.68 | 1.92 | 68.17 | 1.18 | 1.79 | 1.04 | 0.33 |
|  | 39h | 0.43 | 0.88 | 1.85 | 2.18 | 0.65 | 1.31 | 0.27 | 13.60 | 0.00 | 0.17 | 4.60 | 0.64 | 0.44 | 0.24 | 1.70 | 4.08 | 1.91 | 52.23 | 0.00 | 1.36 | 1.12 | 0.36 |
|  | 43h | 0.45 | 0.93 | 2.04 | 2.26 | 0.93 | 1.63 | 0.31 | 14.00 | 0.00 | 0.15 | 7.48 | 0.71 | 0.49 | 0.28 | 1.53 | 8.47 | 1.65 | 49.99 | 0.00 | 1.34 | 1.14 | 0.38 |
|  | 63h | 0.38 | 0.98 | 2.78 | 2.33 | 1.51 | 2.68 | 0.45 | 15.91 | 0.20 | 0.15 | 7.60 | 0.88 | 0.83 | 0.37 | 1.90 | 10.65 | 1.19 | 54.44 | 0.00 | 0.99 | 0.77 | 0.40 |
|  | 66h | 0.32 | 0.89 | 2.71 | 2.08 | 1.46 | 2.60 | 0.39 | 16.67 | 0.00 | 0.00 | 7.67 | 0.93 | 0.86 | 0.37 | 2.15 | 11.78 | 1.41 | 55.13 | 0.00 | 0.90 | 0.66 | 0.40 |
| Aniosrub | 0h | 0.00 | 2.19 | 2.22 | 2.68 | 0.00 | 0.00 | 0.00 | 8.48 | 0.00 | 0.00 | 0.00 | 0.00 | 0.00 | 0.00 | 4.39 | 2.50 | 2.26 | 75.28 | 0.00 | 0.00 | 0.00 | 0.22 |
|  | 15h | 0.57 | 1.16 | 1.58 | 2.76 | 0.16 | 0.00 | 0.00 | 10.67 | 0.00 | 0.17 | 0.00 | 0.26 | 0.00 | 0.20 | 4.29 | 2.36 | 1.73 | 68.92 | 1.14 | 1.88 | 1.05 | 0.31 |
|  | 18h | 0.67 | 1.91 | 1.33 | 3.02 | 0.00 | 0.00 | 0.00 | 11.56 | 0.00 | 0.00 | 0.00 | 0.00 | 0.00 | 0.00 | 4.89 | 3.05 | 1.76 | 65.01 | 0.00 | 2.12 | 0.00 | 0.32 |
|  | 39h | 0.34 | 0.59 | 1.88 | 1.68 | 0.38 | 10.29 | 1.84 | 13.98 | 1.31 | 0.00 | 0.00 | 0.36 | 5.23 | 1.29 | 1.55 | 2.83 | 1.04 | 51.13 | 0.00 | 0.80 | 0.73 | 0.38 |
|  | 43h | 0.53 | 0.64 | 2.06 | 1.86 | 0.39 | 12.67 | 2.08 | 14.33 | 1.38 | 0.00 | 0.00 | 0.35 | 5.51 | 1.39 | 1.48 | 2.60 | 0.86 | 45.77 | 0.00 | 0.73 | 0.66 | 0.44 |
|  | 63h | 0.46 | 0.49 | 2.15 | 1.26 | 0.61 | 27.16 | 2.59 | 13.00 | 1.59 | 0.00 | 0.00 | 0.29 | 7.04 | 1.19 | 1.35 | 1.52 | 0.71 | 34.62 | 0.00 | 0.52 | 0.53 | 0.53 |
|  | 66h | 0.53 | 0.41 | 2.16 | 1.43 | 0.61 | 27.01 | 2.53 | 12.65 | 1.59 | 0.00 | 0.00 | 0.29 | 6.96 | 1.13 | 1.14 | 1.49 | 0.69 | 34.56 | 0.00 | 0.52 | 0.53 | 0.51 |

**Table S4.** Fatty acid composition, and respective calculated degree of saturation (Deg Sat), of non-adapted cells of *M. vaccae* and previously adapted cells to alcohol gel (Alc gel) and Aniosrub (Anios), when exposed to the disinfectants, antibiotics, and efflux pump inhibitors. The antibiotics tested were levofloxacin (Levo) and teicoplanin (Teico), and the efflux pump inhibitors were thioridazine (Thio), and omeprazole (Ome).

|  | **Disinfectant** | **Time** | **10:0** | **12:0** | **14:0** | **14:1**  **w9c** | **15:0** | **15:0**  ***iso*** | **15:0**  ***anteiso*** | **16:0** | **16:0**  ***iso*** | **16:0**  ***anteiso*** | **16:1**  **w6c** | **17:0** | **17:0**  ***iso*** | **17:0**  ***anteiso*** | **18:0** | **18:0**  **10-methyl** | **18:1**  **w7c** | **18:1**  **w9c** | **20:0** | **22:0** | **24:0** | **Deg Sat** |
| --- | --- | --- | --- | --- | --- | --- | --- | --- | --- | --- | --- | --- | --- | --- | --- | --- | --- | --- | --- | --- | --- | --- | --- | --- |
| Not adapted | Alcohol gel | 2h | 0.58 | 1.53 | 1.47 | 1.47 | 0.14 | 1.96 | 2.66 | 8.91 | 0.18 | 0.09 | 4.39 | 0.24 | 1.15 | 0.98 | 5.54 | 2.59 | 2.96 | 54.94 | 1.33 | 1.09 | 0.68 | 0.34 |
|  |  | 16h | 0.33 | 1.03 | 1.73 | 0.94 | 0.40 | 7.26 | 6.63 | 7.52 | 0.63 | 0.00 | 4.23 | 0.49 | 2.37 | 1.37 | 4.93 | 2.24 | 4.19 | 43.42 | 1.69 | 0.75 | 0.50 | 0.37 |
|  |  | 40h | 0.23 | 1.02 | 1.80 | 0.86 | 1.16 | 9.81 | 3.92 | 7.12 | 1.42 | 0.00 | 3.87 | 1.24 | 3.66 | 1.14 | 4.60 | 2.06 | 3.24 | 37.75 | 1.13 | 1.04 | 0.52 | 0.43 |
|  |  | 70h | 0.30 | 1.30 | 1.50 | 0.86 | 0.88 | 10.91 | 3.42 | 7.70 | 1.05 | 0.00 | 3.34 | 0.80 | 5.38 | 1.03 | 3.00 | 4.28 | 2.39 | 40.19 | 0.54 | 0.57 | 0.57 | 0.37 |
|  | Alc gel + Levo | 2h | 0.57 | 1.74 | 1.54 | 1.88 | 0.23 | 2.32 | 0.46 | 8.16 | 0.45 | 0.11 | 4.84 | 0.31 | 1.65 | 0.32 | 4.15 | 4.08 | 1.84 | 56.23 | 0.71 | 1.39 | 1.00 | 0.31 |
|  |  | 16h | 0.89 | 2.09 | 1.66 | 2.72 | 0.36 | 0.84 | 0.53 | 7.79 | 0.00 | 0.00 | 0.00 | 0.37 | 0.46 | 0.25 | 2.16 | 4.29 | 1.03 | 65.96 | 0.57 | 1.41 | 1.17 | 0.26 |
|  |  | 40h | 0.94 | 2.09 | 1.59 | 2.80 | 0.31 | 0.75 | 0.53 | 8.65 | 0.00 | 0.12 | 0.00 | 0.33 | 0.61 | 0.33 | 1.84 | 5.75 | 0.81 | 63.73 | 0.39 | 1.40 | 1.11 | 0.28 |
|  |  | 70h | 1.00 | 2.10 | 1.52 | 2.88 | 0.26 | 0.65 | 0.54 | 9.52 | 0.00 | 0.23 | 0.00 | 0.29 | 0.76 | 0.42 | 1.52 | 7.21 | 0.59 | 61.51 | 0.21 | 1.40 | 1.04 | 0.29 |
|  | Alc gel + Teico | 2h | 0.54 | 1.64 | 1.54 | 1.87 | 0.18 | 2.27 | 0.63 | 7.99 | 0.45 | 0.00 | 4.73 | 0.30 | 1.64 | 0.34 | 4.18 | 4.08 | 1.86 | 57.48 | 0.75 | 1.34 | 0.90 | 0.29 |
|  |  | 16h | 0.58 | 1.83 | 1.52 | 2.83 | 0.25 | 1.59 | 0.39 | 7.46 | 0.23 | 0.00 | 0.00 | 0.30 | 1.57 | 0.28 | 2.16 | 3.10 | 0.95 | 68.48 | 0.30 | 1.09 | 0.79 | 0.23 |
|  |  | 40h | 0.24 | 0.66 | 1.85 | 0.80 | 0.20 | 0.51 | 0.12 | 17.47 | 0.07 | 0.00 | 0.00 | 0.28 | 0.84 | 0.12 | 0.85 | 0.91 | 0.36 | 53.14 | 0.00 | 0.31 | 0.25 | 0.41 |
|  |  | 70h | 0.00 | 0.58 | 2.02 | 0.22 | 0.45 | 0.21 | 0.07 | 26.36 | 0.09 | 0.00 | 0.00 | 0.77 | 0.59 | 0.09 | 1.26 | 0.88 | 0.31 | 41.42 | 0.00 | 0.00 | 0.23 | 0.75 |
|  | Alc gel + Thio | 2h | 0.64 | 1.81 | 1.48 | 1.95 | 0.20 | 2.07 | 0.53 | 8.18 | 0.41 | 0.00 | 4.64 | 0.32 | 1.70 | 0.34 | 4.30 | 3.94 | 2.33 | 55.96 | 0.73 | 1.50 | 1.03 | 0.31 |
|  |  | 16h | 0.83 | 1.53 | 2.83 | 1.85 | 0.54 | 5.69 | 1.06 | 7.51 | 0.62 | 0.06 | 0.00 | 0.29 | 1.88 | 0.58 | 2.31 | 2.32 | 1.68 | 57.65 | 0.28 | 0.69 | 0.54 | 0.28 |
|  |  | 40h | 0.36 | 1.11 | 3.28 | 1.51 | 0.45 | 9.90 | 1.93 | 8.81 | 1.13 | 0.00 | 0.00 | 0.31 | 3.56 | 0.56 | 1.91 | 1.62 | 1.10 | 53.23 | 0.34 | 0.83 | 0.60 | 0.32 |
|  |  | 70h | 0.33 | 0.73 | 2.01 | 0.33 | 0.82 | 24.82 | 3.92 | 11.92 | 2.55 | 0.00 | 0.75 | 0.26 | 5.66 | 0.40 | 0.97 | 0.74 | 0.61 | 20.50 | 0.24 | 0.38 | 0.40 | 0.81 |
|  | Alc gel + Ome | 2h | 0.49 | 1.70 | 1.61 | 1.58 | 0.27 | 2.40 | 2.41 | 9.26 | 0.26 | 0.00 | 4.44 | 0.28 | 1.33 | 0.91 | 5.09 | 2.88 | 2.89 | 54.02 | 1.27 | 1.19 | 0.75 | 0.35 |
|  |  | 16h | 0.61 | 1.27 | 1.77 | 1.46 | 0.61 | 7.11 | 6.05 | 8.79 | 1.50 | 0.00 | 3.67 | 0.27 | 3.97 | 1.90 | 3.13 | 2.04 | 2.77 | 42.91 | 1.20 | 0.69 | 0.52 | 0.37 |
|  |  | 40h | 0.22 | 0.89 | 1.78 | 0.92 | 0.84 | 10.41 | 6.58 | 9.81 | 2.04 | 0.00 | 2.68 | 0.31 | 5.78 | 2.25 | 2.91 | 1.82 | 2.67 | 37.48 | 1.08 | 0.45 | 0.35 | 0.43 |
|  |  | 70h | 0.00 | 1.32 | 1.36 | 0.00 | 1.53 | 13.23 | 5.74 | 8.49 | 1.35 | 0.00 | 1.59 | 0.32 | 8.92 | 1.84 | 3.50 | 2.36 | 2.52 | 32.08 | 0.86 | 0.00 | 0.00 | 0.48 |
| Not adapted | Aniosrub | 2h | 0.39 | 1.16 | 1.26 | 0.86 | 0.00 | 0.00 | 0.00 | 11.15 | 0.00 | 0.00 | 8.64 | 0.00 | 0.00 | 0.00 | 2.67 | 8.06 | 2.13 | 61.68 | 0.00 | 1.16 | 0.83 | 0.25 |
|  |  | 16h | 3.46 | 1.18 | 1.09 | 6.56 | 0.00 | 4.54 | 0.00 | 7.62 | 0.00 | 0.00 | 5.79 | 0.00 | 2.56 | 0.00 | 2.63 | 4.65 | 2.20 | 61.03 | 0.00 | 0.86 | 0.62 | 0.23 |
|  |  | 40h | 1.37 | 1.00 | 1.23 | 6.65 | 0.00 | 11.23 | 0.34 | 11.03 | 2.92 | 0.00 | 2.41 | 0.00 | 9.25 | 0.30 | 1.95 | 2.24 | 1.00 | 33.53 | 0.75 | 0.41 | 0.00 | 0.41 |
|  |  | 70h | 0.60 | 0.29 | 1.51 | 3.89 | 0.66 | 18.29 | 0.29 | 11.48 | 4.91 | 0.00 | 1.93 | 0.42 | 12.50 | 0.47 | 2.00 | 1.22 | 0.93 | 27.85 | 0.35 | 0.00 | 0.00 | 0.50 |
|  | Anios + Levo | 2h | 0.00 | 1.22 | 1.49 | 0.88 | 0.00 | 0.00 | 0.00 | 11.29 | 0.00 | 0.00 | 8.79 | 0.00 | 0.00 | 0.00 | 2.63 | 8.15 | 2.08 | 61.48 | 0.00 | 1.17 | 0.81 | 0.25 |
|  |  | 16h | 3.92 | 1.15 | 1.04 | 5.94 | 0.00 | 0.00 | 0.00 | 7.65 | 0.00 | 0.00 | 5.78 | 0.00 | 0.00 | 0.00 | 2.64 | 5.11 | 2.16 | 59.62 | 0.00 | 0.91 | 0.59 | 0.24 |
|  |  | 40h | 3.60 | 1.10 | 1.02 | 6.44 | 0.00 | 0.00 | 0.00 | 7.26 | 0.00 | 0.00 | 5.12 | 0.00 | 0.00 | 0.00 | 2.98 | 4.46 | 2.36 | 59.45 | 0.00 | 0.82 | 0.52 | 0.24 |
|  |  | 70h | 3.36 | 1.00 | 0.94 | 5.65 | 0.00 | 0.00 | 0.00 | 7.63 | 0.00 | 0.00 | 4.72 | 0.00 | 0.00 | 0.00 | 10.06 | 4.09 | 2.19 | 53.77 | 0.38 | 0.49 | 0.00 | 0.36 |
|  | Anios + Teico | 2h | 0.00 | 1.38 | 1.52 | 0.92 | 0.00 | 0.00 | 0.00 | 11.49 | 0.00 | 0.00 | 8.95 | 0.00 | 0.00 | 0.00 | 2.53 | 8.44 | 2.03 | 61.13 | 0.00 | 0.98 | 0.64 | 0.25 |
|  |  | 16h | 0.34 | 1.22 | 1.11 | 0.63 | 0.00 | 0.00 | 0.00 | 12.29 | 0.00 | 0.00 | 5.75 | 0.00 | 0.00 | 0.00 | 2.63 | 4.87 | 2.17 | 58.83 | 0.00 | 0.70 | 0.44 | 0.28 |
|  |  | 40h | 0.57 | 0.65 | 1.29 | 1.02 | 0.07 | 0.14 | 0.00 | 27.06 | 0.00 | 0.00 | 4.85 | 0.18 | 0.00 | 0.00 | 1.20 | 3.21 | 2.83 | 48.23 | 0.00 | 0.00 | 0.00 | 0.54 |
|  |  | 70h | 0.41 | 0.27 | 1.62 | 0.79 | 0.09 | 0.41 | 0.00 | 32.33 | 0.00 | 0.00 | 2.21 | 0.17 | 0.00 | 0.00 | 1.47 | 1.54 | 2.28 | 40.29 | 0.00 | 0.00 | 0.00 | 0.80 |
|  | Anios + Thio | 2h | 0.00 | 1.41 | 1.70 | 5.62 | 0.00 | 0.00 | 0.00 | 8.21 | 0.00 | 0.00 | 10.61 | 0.00 | 0.00 | 0.00 | 2.93 | 10.45 | 1.89 | 61.35 | 0.00 | 1.12 | 0.00 | 0.19 |
|  |  | 16h | 3.97 | 1.25 | 1.24 | 3.87 | 0.00 | 0.00 | 0.00 | 8.78 | 0.00 | 0.00 | 6.83 | 0.00 | 0.00 | 0.00 | 3.04 | 6.04 | 2.03 | 59.04 | 0.00 | 0.80 | 0.00 | 0.27 |
|  |  | 40h | 3.51 | 1.27 | 1.19 | 3.65 | 0.00 | 0.00 | 0.00 | 8.96 | 0.00 | 0.00 | 6.85 | 0.00 | 0.00 | 0.00 | 2.91 | 5.27 | 2.89 | 55.48 | 0.00 | 0.73 | 0.00 | 0.27 |
|  |  | 70h | 4.89 | 0.25 | 1.17 | 0.49 | 0.00 | 0.00 | 0.00 | 29.98 | 0.00 | 0.00 | 1.52 | 0.15 | 0.00 | 0.00 | 1.57 | 1.70 | 2.68 | 44.30 | 0.00 | 0.00 | 0.00 | 0.78 |
|  | Anios + Ome | 2h | 0.00 | 1.34 | 1.57 | 2.47 | 0.00 | 0.00 | 0.00 | 10.33 | 0.00 | 0.00 | 9.45 | 0.00 | 0.00 | 0.00 | 2.69 | 9.01 | 2.00 | 61.32 | 0.00 | 1.09 | 0.48 | 0.23 |
|  |  | 16h | 0.00 | 1.23 | 1.60 | 0.95 | 0.00 | 0.00 | 0.00 | 11.90 | 0.00 | 0.00 | 9.13 | 0.00 | 0.00 | 0.00 | 2.51 | 8.59 | 1.86 | 60.65 | 0.00 | 0.96 | 0.64 | 0.26 |
|  |  | 40h | 1.83 | 1.02 | 1.05 | 1.75 | 0.00 | 0.00 | 0.00 | 14.05 | 0.00 | 0.00 | 5.54 | 0.00 | 0.00 | 0.00 | 2.28 | 4.83 | 2.08 | 58.24 | 0.00 | 0.67 | 0.00 | 0.31 |
|  |  | 70h | 1.12 | 0.60 | 1.60 | 1.99 | 0.00 | 0.00 | 0.00 | 20.39 | 0.00 | 0.00 | 0.00 | 0.00 | 0.00 | 0.00 | 2.42 | 1.39 | 1.95 | 44.81 | 0.00 | 0.00 | 0.00 | 0.54 |
| Adapted to Alcohol gel | Alcohol gel | 2h | 0.58 | 1.00 | 3.96 | 0.95 | 0.80 | 3.18 | 0.71 | 6.81 | 0.31 | 0.00 | 2.13 | 0.49 | 0.62 | 0.40 | 2.10 | 3.29 | 1.53 | 66.08 | 0.00 | 0.00 | 0.00 | 0.22 |
|  |  | 16h | 0.00 | 0.61 | 4.70 | 0.80 | 0.91 | 11.60 | 1.56 | 10.78 | 2.62 | 0.00 | 0.00 | 0.38 | 4.17 | 0.86 | 2.22 | 2.55 | 2.38 | 49.43 | 0.00 | 0.00 | 0.00 | 0.37 |
|  |  | 40h | 0.37 | 0.57 | 4.61 | 0.79 | 0.88 | 11.65 | 1.58 | 10.69 | 2.58 | 0.00 | 0.00 | 0.37 | 4.16 | 0.83 | 2.10 | 1.82 | 2.34 | 48.08 | 0.00 | 0.00 | 0.00 | 0.38 |
|  |  | 70h | 0.17 | 1.64 | 3.11 | 2.65 | 1.63 | 13.22 | 0.47 | 12.99 | 2.85 | 0.00 | 0.87 | 0.69 | 5.83 | 0.00 | 4.65 | 0.89 | 1.58 | 56.47 | 0.00 | 0.00 | 0.00 | 0.40 |
|  | Alc gel + Levo | 2h | 0.81 | 1.42 | 3.00 | 2.42 | 1.66 | 2.92 | 0.00 | 12.37 | 0.00 | 0.00 | 5.52 | 0.67 | 0.75 | 0.00 | 3.79 | 8.05 | 1.55 | 51.88 | 0.00 | 0.00 | 0.00 | 0.39 |
|  |  | 16h | 0.89 | 1.75 | 3.26 | 2.70 | 1.78 | 3.04 | 0.00 | 12.87 | 0.00 | 0.00 | 5.66 | 0.70 | 0.87 | 0.00 | 3.66 | 6.86 | 1.67 | 57.58 | 0.00 | 0.00 | 0.00 | 0.37 |
|  |  | 40h | 1.81 | 1.43 | 3.01 | 1.76 | 1.57 | 2.60 | 0.86 | 12.31 | 0.00 | 0.00 | 5.64 | 1.10 | 0.85 | 0.00 | 3.20 | 5.66 | 1.12 | 41.19 | 0.00 | 0.00 | 0.83 | 0.51 |
|  |  | 70h | 0.23 | 1.58 | 2.91 | 2.67 | 1.55 | 5.73 | 0.48 | 12.02 | 0.00 | 0.00 | 5.62 | 0.66 | 0.69 | 0.00 | 3.73 | 8.02 | 1.96 | 51.81 | 0.00 | 0.00 | 0.00 | 0.37 |
|  | Alc gel + Teico | 2h | 0.99 | 1.66 | 2.99 | 2.41 | 1.63 | 2.93 | 0.00 | 11.91 | 0.00 | 0.00 | 5.29 | 0.67 | 0.79 | 0.00 | 3.83 | 8.30 | 1.75 | 52.07 | 0.00 | 0.00 | 0.00 | 0.38 |
|  |  | 16h | 1.34 | 1.76 | 3.27 | 2.80 | 1.78 | 3.07 | 0.00 | 12.65 | 0.00 | 0.00 | 5.81 | 0.69 | 0.85 | 0.00 | 3.20 | 7.97 | 1.66 | 57.98 | 0.00 | 0.00 | 0.00 | 0.36 |
|  |  | 40h | 0.77 | 1.67 | 2.93 | 2.36 | 1.54 | 2.95 | 0.65 | 11.21 | 0.00 | 0.00 | 4.76 | 0.64 | 0.79 | 0.00 | 3.21 | 7.65 | 1.98 | 54.73 | 0.00 | 0.00 | 0.00 | 0.34 |
|  |  | 70h | 2.09 | 1.17 | 2.20 | 1.31 | 1.28 | 2.24 | 0.00 | 10.33 | 0.00 | 0.00 | 4.41 | 0.60 | 0.67 | 0.00 | 4.58 | 5.21 | 3.92 | 63.21 | 0.00 | 0.00 | 0.00 | 0.31 |
|  | Alc gel + Thio | 2h | 1.26 | 1.08 | 1.89 | 1.61 | 1.11 | 2.11 | 0.71 | 10.14 | 0.00 | 0.00 | 3.80 | 0.68 | 0.64 | 0.00 | 4.33 | 7.12 | 3.74 | 55.29 | 0.00 | 0.00 | 0.00 | 0.32 |
|  |  | 16h | 1.06 | 1.51 | 2.49 | 2.22 | 1.34 | 2.48 | 0.00 | 10.50 | 0.00 | 0.00 | 4.23 | 0.00 | 0.66 | 0.00 | 4.42 | 6.61 | 2.76 | 54.60 | 0.00 | 0.00 | 0.00 | 0.33 |
|  |  | 40h | 2.20 | 1.08 | 2.43 | 1.33 | 1.34 | 3.21 | 0.55 | 9.27 | 0.00 | 0.00 | 3.28 | 0.53 | 0.73 | 0.00 | 3.96 | 5.43 | 3.57 | 57.97 | 0.00 | 0.00 | 0.00 | 0.31 |
|  |  | 70h | 0.00 | 0.71 | 1.33 | 1.11 | 0.72 | 1.34 | 0.00 | 5.28 | 0.00 | 0.00 | 2.46 | 0.31 | 0.42 | 0.00 | 1.38 | 3.59 | 0.72 | 22.57 | 0.00 | 0.00 | 0.00 | 0.36 |
|  | Alc gel + Ome | 2h | 0.36 | 0.65 | 1.48 | 1.09 | 0.71 | 1.24 | 0.00 | 5.52 | 0.00 | 0.00 | 2.37 | 0.30 | 0.31 | 0.00 | 1.49 | 3.53 | 0.73 | 25.77 | 0.00 | 0.00 | 0.00 | 0.35 |
|  |  | 16h | 0.32 | 0.29 | 1.89 | 0.42 | 0.50 | 5.66 | 0.63 | 3.96 | 1.08 | 0.00 | 0.79 | 0.25 | 2.42 | 0.34 | 1.50 | 2.35 | 1.75 | 30.73 | 0.00 | 0.00 | 0.00 | 0.26 |
|  |  | 40h | 0.00 | 0.27 | 1.75 | 0.43 | 0.49 | 5.73 | 0.56 | 3.98 | 1.05 | 0.00 | 0.83 | 0.24 | 2.38 | 0.32 | 1.37 | 1.18 | 1.50 | 28.68 | 0.00 | 0.00 | 0.00 | 0.26 |
|  |  | 70h | 0.18 | 0.15 | 1.16 | 0.24 | 0.21 | 12.50 | 1.04 | 3.95 | 0.82 | 0.00 | 0.29 | 0.49 | 2.40 | 0.24 | 0.75 | 0.72 | 0.63 | 16.20 | 0.00 | 0.00 | 0.00 | 0.40 |
| Adapted to Aniosrub | Aniosrub | 2h | 4.13 | 1.43 | 1.39 | 1.66 | 0.00 | 6.24 | 1.07 | 12.11 | 0.93 | 0.00 | 4.35 | 0.00 | 4.20 | 0.73 | 15.02 | 1.94 | 1.61 | 40.30 | 0.00 | 0.00 | 0.00 | 0.71 |
|  |  | 16h | 2.77 | 1.73 | 1.54 | 1.55 | 0.00 | 6.95 | 1.23 | 13.23 | 0.95 | 0.00 | 0.00 | 0.00 | 4.64 | 0.00 | 7.92 | 2.30 | 2.01 | 50.37 | 0.00 | 0.00 | 0.00 | 0.50 |
|  |  | 40h | 0.52 | 0.58 | 0.87 | 0.81 | 0.00 | 8.56 | 5.23 | 31.73 | 1.10 | 0.00 | 1.56 | 0.00 | 4.08 |  | 10.38 | 0.68 | 0.57 | 23.26 |  | 0.45 | 0.00 | 1.70 |
|  |  | 70h | 0.57 | 0.40 | 0.58 | 0.46 | 0.00 | 5.10 | 4.29 | 39.37 | 0.76 | 0.00 | 0.00 | 0.00 | 3.52 |  | 13.71 | 0.36 | 0.00 | 16.39 |  | 0.29 | 0.00 | 3.26 |
|  | Anios + Levo | 2h | 4.80 | 1.55 | 1.46 | 2.03 | 0.00 | 6.37 | 2.12 | 11.90 | 0.83 | 0.00 | 4.85 | 0.00 | 4.37 | 1.03 | 7.20 | 2.03 | 1.76 | 43.77 | 0.00 | 0.00 | 0.00 | 0.51 |
|  |  | 16h | 2.88 | 1.77 | 1.64 | 2.05 | 0.00 | 6.68 | 1.46 | 12.89 | 0.84 | 0.00 | 0.00 | 0.00 | 4.49 | 0.00 | 6.46 | 2.28 | 1.99 | 49.93 | 0.00 | 0.00 | 0.00 | 0.48 |
|  |  | 40h | 2.78 | 3.10 | 3.31 | 1.74 | 1.43 | 4.35 | 0.75 | 12.00 | 0.00 | 0.00 | 0.00 | 1.56 | 3.10 | 0.00 | 7.92 | 1.76 | 1.65 | 47.04 | 0.00 | 0.00 | 0.00 | 0.64 |
|  |  | 70h | 1.81 | 1.57 | 2.55 | 1.08 | 0.42 | 2.13 | 0.33 | 26.52 | 0.00 | 0.00 | 0.00 | 0.00 | 1.65 | 0.00 | 3.48 | 1.00 | 1.38 | 36.43 | 0.00 | 0.00 | 0.00 | 0.93 |
|  | Anios + Teico | 2h | 5.87 | 1.81 | 1.60 | 1.99 | 0.00 | 6.39 | 0.96 | 12.35 | 0.77 | 0.00 | 4.89 | 0.00 | 4.32 | 0.00 | 6.42 | 2.03 | 1.66 | 43.65 | 0.00 | 0.00 | 0.00 | 0.54 |
|  |  | 16h | 4.35 | 2.09 | 1.73 | 2.15 | 0.00 | 6.47 | 1.30 | 13.05 | 0.00 | 0.00 | 0.00 | 0.00 | 4.50 | 0.00 | 7.43 | 2.33 | 1.85 | 49.33 | 0.00 | 0.00 | 0.00 | 0.54 |
|  |  | 40h | 3.58 | 2.22 | 1.91 | 2.73 | 0.00 | 4.48 | 0.00 | 13.51 | 0.00 | 0.00 | 0.00 | 0.00 | 4.10 | 0.00 | 6.88 | 2.28 | 1.97 | 48.83 | 0.00 | 0.00 | 0.00 | 0.52 |
|  |  | 70h | 0.00 | 0.00 | 1.62 | 2.89 | 0.00 | 4.31 | 1.80 | 14.33 | 0.00 | 0.00 | 0.00 | 0.00 | 3.57 | 0.00 | 6.35 | 2.20 | 2.01 | 50.53 | 0.00 | 0.00 | 0.00 | 0.40 |
|  | Anios + Thio | 2h | 5.63 | 1.76 | 1.45 | 1.65 | 0.00 | 5.74 | 1.58 | 12.94 | 0.00 | 0.00 | 0.00 | 0.00 | 3.90 | 0.00 | 5.68 | 2.19 | 2.27 | 49.93 | 0.00 | 0.00 | 0.00 | 0.51 |
|  |  | 16h | 4.47 | 2.01 | 1.81 | 2.47 | 0.00 | 5.17 | 0.00 | 14.36 | 0.00 | 0.00 | 0.00 | 0.00 | 3.70 | 0.00 | 9.37 | 2.56 | 1.98 | 49.65 | 0.00 | 0.00 | 0.00 | 0.59 |
|  |  | 40h | 4.52 | 2.14 | 1.76 | 1.58 | 0.00 | 3.78 | 0.99 | 12.61 | 0.00 | 0.00 | 0.00 | 0.00 | 3.03 | 0.00 | 7.60 | 2.02 | 1.89 | 41.77 | 0.00 | 0.00 | 0.00 | 0.63 |
|  |  | 70h | 0.00 | 1.97 | 1.72 | 2.34 | 0.00 | 4.05 | 1.28 | 14.53 | 0.00 | 0.00 | 0.00 | 0.00 | 2.83 | 0.00 | 11.95 | 1.98 | 1.58 | 38.62 | 0.00 | 0.00 | 0.00 | 0.71 |
|  | Anios + Ome | 2h | 1.21 | 0.37 | 0.37 | 0.13 | 0.15 | 0.68 | 0.10 | 1.36 | 0.09 | 0.00 | 0.00 | 0.16 | 0.46 | 0.07 | 0.76 | 0.18 | 0.17 | 4.11 | 0.27 | 0.00 | 0.00 | 1.05 |
|  |  | 16h | 1.05 | 0.22 | 0.20 | 0.00 | 0.00 | 0.76 | 0.11 | 1.48 | 0.10 | 0.00 | 0.00 | 0.00 | 0.52 | 0.00 | 0.78 | 0.22 | 0.21 | 5.08 | 0.23 | 0.00 | 0.00 | 0.75 |
|  |  | 40h | 0.49 | 0.30 | 2.98 | 0.14 | 0.40 | 7.18 | 0.30 | 5.87 | 1.80 | 0.00 | 0.29 | 0.21 | 2.38 | 1.00 | 0.93 | 0.00 | 0.64 | 30.09 | 0.22 | 0.00 | 0.00 | 0.37 |
|  |  | 70h | 0.61 | 0.21 | 1.13 | 0.00 | 0.25 | 4.75 | 0.43 | 3.20 | 0.92 | 0.00 | 0.23 | 0.00 | 1.65 | 1.10 | 1.48 | 0.00 | 0.57 | 16.14 | 0.42 | 0.00 | 0.00 | 0.43 |
